# Supplementary material for: HIVprotI: an integrated web based platform for prediction and design of HIV proteins inhibitors
Source: J Cheminform. 2018 Mar 9;10:12. doi: 10.1186/s13321-018-0266-y (PMC5845081; doi:10.1186/s13321-018-0266-y)
Supplement: Supplementary file 2 — Additional file 2. Source code of HIVProtI web server. [file 13321_2018_266_MOESM2_ESM.zip › HIVprotI_Source-code/hivprotI/weka-3-6-6/doc/weka/classifiers/bayes/net/search/local/LocalScoreSearchAlgorithm.html]

LocalScoreSearchAlgorithm


---


|  |  |  |
| --- | --- | --- |
| **Overview** \| **Package** \| **Class** \| **Tree** \| **Deprecated** \| **Index** \| **Help** \| **Weka's home** | |  |
| **PREV CLASS**   **NEXT CLASS** | **FRAMES**    **NO FRAMES**     **All Classes** |  |
| SUMMARY: NESTED \| FIELD \| CONSTR \| METHOD | DETAIL: FIELD \| CONSTR \| METHOD |  |


---


## weka.classifiers.bayes.net.search.local Class LocalScoreSearchAlgorithm

```
java.lang.Object
  weka.classifiers.bayes.net.search.SearchAlgorithm
      weka.classifiers.bayes.net.search.local.LocalScoreSearchAlgorithm
```

**All Implemented Interfaces:**: java.io.Serializable, OptionHandler, RevisionHandler

**Direct Known Subclasses:**: CISearchAlgorithm, GeneticSearch, HillClimber, K2, SimulatedAnnealing, TAN

---

``` public class LocalScoreSearchAlgorithm extends SearchAlgorithm ```

The ScoreBasedSearchAlgorithm class supports Bayes net structure search algorithms that are based on maximizing scores (as opposed to for example conditional independence based search algorithms).


Valid options are:

```
 -mbc
  Applies a Markov Blanket correction to the network structure, 
  after a network structure is learned. This ensures that all 
  nodes in the network are part of the Markov blanket of the 
  classifier node.
```

```
 -S [BAYES|MDL|ENTROPY|AIC|CROSS_CLASSIC|CROSS_BAYES]
  Score type (BAYES, BDeu, MDL, ENTROPY and AIC)
```

**Version:**
:   $Revision: 5196 $

**Author:**
:   Remco Bouckaert

**See Also:**: Serialized Form

---

| **Field Summary** | |
| --- | --- |
| `static Tag[]` | `TAGS_SCORE_TYPE`             the score types |


**Constructor Summary**

`LocalScoreSearchAlgorithm()`
  
          default constructor

`LocalScoreSearchAlgorithm(BayesNet bayesNet,
Instances instances)`
  
          constructor


| **Method Summary** | |
| --- | --- |
| `void` | `buildStructure(BayesNet bayesNet, Instances instances)`             buildStructure determines the network structure/graph of the network with the K2 algorithm, restricted by its initial structure (which can be an empty graph, or a Naive Bayes graph. |
| `double` | `calcNodeScore(int nNode)`             Calc Node Score for given parent set |
| `double` | `calcScoreWithExtraParent(int nNode, int nCandidateParent)`             Calc Node Score With AddedParent |
| `double` | `calcScoreWithMissingParent(int nNode, int nCandidateParent)`             Calc Node Score With Parent Deleted |
| `boolean` | `getMarkovBlanketClassifier()` |
| `java.lang.String[]` | `getOptions()`             Gets the current settings of the search algorithm. |
| `java.lang.String` | `getRevision()`             Returns the revision string. |
| `SelectedTag` | `getScoreType()`             get quality measure to be used in searching for networks. |
| `java.lang.String` | `globalInfo()`             This will return a string describing the search algorithm. |
| `java.util.Enumeration` | `listOptions()`             Returns an enumeration describing the available options |
| `double` | `logScore(int nType)`             logScore returns the log of the quality of a network (e.g. |
| `java.lang.String` | `markovBlanketClassifierTipText()` |
| `java.lang.String` | `scoreTypeTipText()` |
| `void` | `setMarkovBlanketClassifier(boolean bMarkovBlanketClassifier)` |
| `void` | `setOptions(java.lang.String[] options)`             Parses a given list of options. |
| `void` | `setScoreType(SelectedTag newScoreType)`             set quality measure to be used in searching for networks. |

**Methods inherited from class weka.classifiers.bayes.net.search.SearchAlgorithm**

`initAsNaiveBayesTipText, maxNrOfParentsTipText, toString`

**Methods inherited from class java.lang.Object**

`equals, getClass, hashCode, notify, notifyAll, wait, wait, wait`

**Field Detail**

### TAGS\_SCORE\_TYPE

```
public static final Tag[] TAGS_SCORE_TYPE
```

:   the score types


**Constructor Detail**

### LocalScoreSearchAlgorithm

```
public LocalScoreSearchAlgorithm()
```

:   default constructor

---


### LocalScoreSearchAlgorithm

```
public LocalScoreSearchAlgorithm(BayesNet bayesNet,
                                 Instances instances)
```

:   constructor

    **Parameters:**: `bayesNet` - the network: `instances` - the data


**Method Detail**

### logScore

```
public double logScore(int nType)
```

:   logScore returns the log of the quality of a network
    (e.g. the posterior probability of the network, or the MDL
    value).

    :   **Parameters:**: `nType` - score type (Bayes, MDL, etc) to calculate score with **Returns:**: log score.

---


### buildStructure

```
public void buildStructure(BayesNet bayesNet,
                           Instances instances)
                    throws java.lang.Exception
```

:   buildStructure determines the network structure/graph of the network
    with the K2 algorithm, restricted by its initial structure (which can
    be an empty graph, or a Naive Bayes graph.

    :   **Overrides:**: `buildStructure` in class `SearchAlgorithm`
    :   **Parameters:**: `bayesNet` - the network: `instances` - the data to use **Throws:**: `java.lang.Exception` - if something goes wrong

---


### calcNodeScore

```
public double calcNodeScore(int nNode)
```

:   Calc Node Score for given parent set

    :   **Parameters:**: `nNode` - node for which the score is calculate **Returns:**: log score

---


### calcScoreWithExtraParent

```
public double calcScoreWithExtraParent(int nNode,
                                       int nCandidateParent)
```

:   Calc Node Score With AddedParent

    :   **Parameters:**: `nNode` - node for which the score is calculate: `nCandidateParent` - candidate parent to add to the existing parent set **Returns:**: log score

---


### calcScoreWithMissingParent

```
public double calcScoreWithMissingParent(int nNode,
                                         int nCandidateParent)
```

:   Calc Node Score With Parent Deleted

    :   **Parameters:**: `nNode` - node for which the score is calculate: `nCandidateParent` - candidate parent to delete from the existing parent set **Returns:**: log score

---


### setScoreType

```
public void setScoreType(SelectedTag newScoreType)
```

:   set quality measure to be used in searching for networks.

    :   **Parameters:**: `newScoreType` - the new score type

---


### getScoreType

```
public SelectedTag getScoreType()
```

:   get quality measure to be used in searching for networks.

    :   **Returns:**: quality measure

---


### setMarkovBlanketClassifier

```
public void setMarkovBlanketClassifier(boolean bMarkovBlanketClassifier)
```

:   **Parameters:**: `bMarkovBlanketClassifier` -

---


### getMarkovBlanketClassifier

```
public boolean getMarkovBlanketClassifier()
```

:   **Returns:**

---


### listOptions

```
public java.util.Enumeration listOptions()
```

:   Returns an enumeration describing the available options

    :   **Specified by:**: `listOptions` in interface `OptionHandler` **Overrides:**: `listOptions` in class `SearchAlgorithm`
    :   **Returns:**: an enumeration of all the available options

---


### setOptions

```
public void setOptions(java.lang.String[] options)
                throws java.lang.Exception
```

:   Parses a given list of options.


    Valid options are:

    ```
     -mbc
      Applies a Markov Blanket correction to the network structure, 
      after a network structure is learned. This ensures that all 
      nodes in the network are part of the Markov blanket of the 
      classifier node.
    ```

    ```
     -S [BAYES|MDL|ENTROPY|AIC|CROSS_CLASSIC|CROSS_BAYES]
      Score type (BAYES, BDeu, MDL, ENTROPY and AIC)
    ```

    :   **Specified by:**: `setOptions` in interface `OptionHandler` **Overrides:**: `setOptions` in class `SearchAlgorithm`
    :   **Parameters:**: `options` - the list of options as an array of strings **Throws:**: `java.lang.Exception` - if an option is not supported

---


### getOptions

```
public java.lang.String[] getOptions()
```

:   Gets the current settings of the search algorithm.

    :   **Specified by:**: `getOptions` in interface `OptionHandler` **Overrides:**: `getOptions` in class `SearchAlgorithm`
    :   **Returns:**: an array of strings suitable for passing to setOptions

---


### scoreTypeTipText

```
public java.lang.String scoreTypeTipText()
```

:   **Returns:**: a string to describe the ScoreType option.

---


### markovBlanketClassifierTipText

```
public java.lang.String markovBlanketClassifierTipText()
```

:   **Returns:**: a string to describe the MarkovBlanketClassifier option.

---


### globalInfo

```
public java.lang.String globalInfo()
```

:   This will return a string describing the search algorithm.

    :   **Returns:**: The string.

---


### getRevision

```
public java.lang.String getRevision()
```

:   Returns the revision string.

    :   **Specified by:**: `getRevision` in interface `RevisionHandler` **Overrides:**: `getRevision` in class `SearchAlgorithm`
    :   **Returns:**: the revision


---


|  |  |  |
| --- | --- | --- |
| **Overview** \| **Package** \| **Class** \| **Tree** \| **Deprecated** \| **Index** \| **Help** \| **Weka's home** | |  |
| **PREV CLASS**   **NEXT CLASS** | **FRAMES**    **NO FRAMES**     **All Classes** |  |
| SUMMARY: NESTED \| FIELD \| CONSTR \| METHOD | DETAIL: FIELD \| CONSTR \| METHOD |  |


---
